# Editorial for a New Section: Nutrition and Neuro Sciences

**DOI:** 10.3390/nu17091399

**Published:** 2025-04-22

**Authors:** Lorena Perrone, William B. Grant

**Affiliations:** 1 Department of Life Science, Health and Health Professions, Link Campus University, 00165 Rome, Italy; 2 Sunlight, Nutrition, and Health Research Center, 1745 Pacific Ave., Suite 504, San Francisco, CA 94109, USA

In December 2024, *Nutrients* launched a new section entitled “Nutrition and Neuro Sciences”, with the scope of collecting review and research articles analyzing the impact of nutrition on cognitive function and brain physiology throughout life. This section will focus on interdisciplinary studies that provide new insights into the effect of dietary habits and/or specific nutrients on healthy brain function, promoting healthy aging.

In order to provide examples of studies responding to the aims of this new section of *Nutrients*, we briefly describe the results of the first eight review and research articles published in this new section below while also providing the research context at the basis of these studies and underlining their innovative results and conclusions.

It has been clearly demonstrated that stress exerts a significant effect on the functioning of the entire human body [1]. Chronic stress strongly affects quality of life by enhancing the risk of chronic diseases such as cardiovascular diseases, and exhibits a negative impact on mental and cognitive function by promoting anxiety and depression [2,3]. Thus, identifying effective interventions capable of inhibiting these deleterious effects of chronic stress is vital. Natural products have been shown to be a beneficial therapeutic strategy in this regard. In particular, extracts from the *Tremella fuciformis* (TF) fungus have been employed for many years in traditional medicine, and several studies have suggested that they have beneficial effects against chronic stress-induced illnesses [4] and exert beneficial effects on neuronal cells [5].

In a further study, Moon and colleagues investigated the role of TF enzymatic extracts in preventing the effects promoted by chronic restraint stress (CRS). The authors employed a mouse model of CRS and analyzed the effects of TF extract on CRS-induced anxiety and depression and on neuroinflammatory biomarkers [6]. They found that TF enzymatic extracts ameliorated CSF-induced anxiety, enhanced the expression of neurotrophic factors, and decreased the production of pro-inflammatory cytokines, showing support for the therapeutic potential of the extract in ameliorating CRS-induced disorders [6].

Recently, the number of people affected by mental illnesses has increased. The COVID-19 pandemic further enhanced the risk of developing mental illness by amplifying stress and anxiety worldwide [7]. Several forms of pharmaceutical treatments for mental disorders exist, but they can lead to side effects [8], and moreover may lead to poor tolerability, highly variable response, or enhanced relapse [9]. For this reason, several studies have analyzed the efficacy of natural compounds as therapeutic or adjuvant agents against mental disorders. Various reports have described the beneficial effects of *Crocus sativus*, also called saffron, on mental illnesses [10]. *Scutellaria baicalensis Georgi* also seems to be beneficial against mental disorders and has been traditionally employed in Chinese medicine [11]. Notably, this natural compound is safe and well tolerated, suggesting that it may be employed as a therapeutic or adjuvant agent for patients with mental illnesses [12].

Dormal and colleagues reported the results of a clinical study investigating the safety and synergistic effects of saffron and *Scutellaria baicalensis Georgi* (scutellaria) supplementation in patients affected by mild–moderate depression [13]. They performed a double-blind, randomized, and placebo-controlled clinical study that included 180 subjects affected by depression who were divided into four subgroups: placebo, saffron only, *Scutellaria* only, and treatment with both natural compounds. This study confirmed the safety and beneficial effects of saffron against mood disorders and demonstrated that *Scutellaria* also exhibited a beneficial effect against depression. Notably, the two natural compounds showed a synergistic effect when combined, with minimal side effects. Although these results demonstrated that these two natural compounds can be employed as therapeutic agents against anxiety and depression, further clinical studies are needed to verify the absence of long-term side effects cause by treatment with these two natural compounds.

Several studies demonstrated that malnutrition indicated a poorer prognosis in stroke patients [14]. Indeed, malnutrition exhibits a negative impact on physical and mental status, affecting the patient’s quality of life and enhancing the presence of comorbidities and the rate of mortality [15]. Thus, it is necessary to make an early diagnosis of malnutrition to set up appropriate therapeutic strategies. However, at present, malnutrition is analyzed using composite scores that are simple to apply but inefficient for the personal and continuous assessment of patients [16]. Conversely, Bioelectrical Impedance Analysis (BIA) and Bioelectrical Impedance Vector Analysis (BIVA) provide more accurate measurements of body composition, alterations in cellular mass, and hydration [17], suggesting that these methods could be employed to assess patient malnutrition. However, the equations employed for the calculation present some limitations and thus more clinical evidence is necessary to verify the efficacy of BIA and BIVA in monitoring the malnutrition state of patients.

Dal Bello and colleagues reported the results of a prospective clinical study investigating whether patient malnutrition, assessed by BIVA at admission, correlated with a worse prognosis of stroke [18]; it was found to correlate with a more negative outcome in stroke patients.

Parkinson’s disease (PD) is a neurodegenerative disease characterized by the presence of Lewi bodies, which are constituted by aggregates of α-synuclein that affect neurons in the basal ganglia [19], resulting in motor disabilities. PD strongly affects the quality of life of patients and its incidence has increased in recent years. PD patients also experience non-motor symptoms which appear early in PD progression [20]. Olfactory impairment is one of the non-motor symptoms of PD and occurs at least four years before the appearance of motor dysfunction [21], suggesting that it can be considered an early diagnostic tool to distinguish PD from other neurodegenerative disorders. Similar considerations are also present for the gustative impairment that early-stage PD patients experience, although its relevance and incidence in PD is still debated [22]. Taste and olfactory impairment in PD can seriously affect the quality of life of these patients because it may lead to diabetes, poor social integration, and nutritional alterations [23]. However, at present, there is no therapeutic strategy available to ameliorate these non-motor symptoms. Indeed, several studies are investigating the effects of nutritional interventions as adjuvants to pharmacological treatment for PD [24].

Alia and colleagues produced a narrative review for the new section of *Nutrients* which summarized current knowledge about the impact of taste and olfactory impairment on nutrition in PD [25]. They explored the role of bitter taste in further promoting dysfunction in PD by enhancing α-synuclein aggregation, demonstrating that taste impairment in PD contributes to altered nutrition that promotes PD progression. They described the relevance of dietary intervention in PD to ameliorate some symptoms and progression.

Migraine is a common neurological disorder that affects more than one billion people worldwide [26]. While pharmaceutical treatments are available [26], the use of non-steroidal anti-inflammatory drugs may result in gastrointestinal bleeding as well as heart and renal failure. Tripants and ergot alkaloids entail a risk of coronary heart disease, cerebrovascular disease, uncontrolled hypertension, and peripheral vascular disease, while gepants may also entail several risks. For those who prefer non-pharmaceutical approaches to migraines, dietary patterns and food groups can be considered.

Tu and colleagues summarized what is known about which foods trigger and which protect against migraines and what is known about the mechanisms involved [27]. Trigger foods include alcoholic drinks, caffeine, chocolate (also a source of caffeine), monosodium glutamate, nitrates and nitrites in processed meats, and tyramine (found in aged cheese, fermented foods, and smoked fish). Several of these compounds or foods have beneficial health effects, especially in smaller doses, and so should not necessarily be avoided. Protective foods or diets include omega-3-rich foods such as fatty ocean fish, the Mediterranean diet, foods containing vitamin D (e.g., fish and meat [28]), carbohydrate-restricted diets, and adequate hydration.

In a 2020 review on the role of diet and nutrition in migraine [29], the authors noted that the quality of the evidence regarding diet and migraine was low since most of the studies were cross-sectional studies, and that most of the intervention studies were related to a decrease in the frequency of migraine attacks and were not double-blind.

Some of the trigger mechanisms included alcohol provoking neurogenic inflammation and inhibiting the metabolism of dopamine in the gut, leading to increased dopamine levels. Several mechanisms were also discussed for caffeine, including urinary magnesium loss and dehydration. As for protective foods/diets, it is well known that omega-3 fatty acids, the Mediterranean diet, and vitamin D reduce inflammation. In comparison to the Western diet, the Mediterranean diet contains more vegetables, fruits, cereals, fish, and olive oil and less meat [30,31]. Anyone who suffers from migraine attacks would benefit from reading this timely review.

Kim and colleagues examined the role of selenium (Se) in episodic memory with respect to apolipoprotein E ε4 (APOE4) [32]. The study involved 156 APOE4-negative and 40 APOER-positive participants. The mean age was 73 ± 6 years with a mean episodic memory score (EMS) of 35 ± 9. Mean Se serum concentration was 112 ± 23 µg/L for APOE-negative and 103 ± 16 µg/L for APOE-positive subjects. In the fully adjusted model, serum Se concentrations were significantly correlated with EMS (β = 0.07 [95% confidence interval (CI), 0.03–0.12]) for APOE4-negative subjects but insignificantly correlated with EMS in APOE4-positive subjects. Similar results were obtained for the Consortium to Establish a Registry for Alzheimer’s neuropsychological battery. This study adds to the literature showing that APOE4 positivity increases the risk of adverse brain health. The mechanisms suggested to explain this finding included neutralizing reactive oxidative species and reducing oxidative stress, thereby reducing the aggregation of amyloid beta and the hyperphosphorylation of tau.

According to a 2023 review, the global number of people with Alzheimer’s disease (AD), prodromal AD, and preclinical AD were estimated to be 32 million, 69 million, and 315 million, respectively. Together, they constituted 416 million people across the AD continuum, or 22% of all people aged 50 or older [33]. Given the devastation caused by AD and the number of people affected, it is imperative to conduct research on ways to reduce risk. Amyloid beta and tau are two of the hallmarks of AD [34], while mitochondrial dysfunction is involved in its progression [35,36,37]. It has been demonstrated that the restoration of mitochondrial function by physical exercise and a diet high in antioxidants, or the use of therapeutic approaches, can delay the onset and slow the progression of AD [35].

Rezaee and colleagues examined the effect of polyphenolic extracts from six varieties of sorghum on the attenuation of amyloid beta-induced phospho-tau levels, total tau levels, and mitochondrial dysfunction in neuroblastoma M17 cells [38]. Significant reductions were found for all sorghum extracts for amyloid beta-induced markers and tau protein levels, in addition to improved mitochondrial membrane potential and adenosine triphosphate levels. Those with stronger effects warrant further investigations in other cell and animal models.

The standard ketogenic diet (KD) contains a very high level of fat (75% of energy) and a very low carbohydrate level (5% of energy) [39]. The KD has helped diabetic patients lower HbA1c and reduce their need for insulin [39]. It also appears to lower low-density lipoprotein cholesterol, raise high-density lipoprotein cholesterol, and lower triglycerides.

A randomized crossover trial of a modified KD was conducted with 21 AD patients [40]. The modified KD had an average macronutrient ratio of 58% fat (26% saturated, 32% non-saturated), 29% protein, 7% fiber, and 6% net carbohydrate by weight. Two 12-week treatment periods were carried out. The treatment effects measured were cognition, (+2 ± 9)/(70), daily function (+3 ± 5)/(65), and quality of life (+3 ± 7)/(34). The treatment effects were small and, judging by the standard deviations, not clinically meaningful.

A review of ketogenic interventions found that they were probably effective at inducing cognitive improvement for mild-to-moderate APOE4-negative AD patients and patients with mild cognitive impairment, but unproven for mild-to-moderate ApoE4-positive AD patients [41].

An article by Gentili and colleagues examined the effect of a novel ketone diester, DAG-MAG-BHB, on inflammasome activation in microglial cells in response to beta-amyloid and low-glucose AD-like conditions [42]. The goal was to determine if the compound could reduce central nervous system stressors, including cerebral hypo-glucose metabolism, hyperinsulinemia, mitochondrial dysfunction, oxidative stress, impairment of neuronal autophagy, hypoxic insults, and neuroinflammation. It was previously demonstrated that enhanced activation of NLRP-3 inflammasome in microglia is linked to AD progression [36]. DAG-MAG-BHB enhanced cell viability, preserved morphological integrity, and maintained elevated Acetyl-CoA and ATP levels under hypoglycemic conditions. It increased ATP production via a ketolytic pathway and inhibited NLRP3 inflammasome activation. Thus, the authors suggested it has the potential to contribute to managing neuroinflammatory diseases, and might therefore be used to obtain some of the benefits of the KD indirectly.

In conclusion, these eight articles fully underline the aims and scope of the new section of *Nutrients*, entitled “Nutrition and Neuro Sciences”.

## References

[1] Yaribeygi H., Panahi Y., Sahraei H., Johnston T.P., Sahebkar A. (2017). The impact of stress on body function: A review. EXCLI J..

[2] Swaab D.F., Bao A.M., Lucassen P.J. (2005). The stress system in the human brain in depression and neurodegeneration. Ageing Res. Rev..

[3] McEwen B.S. (2017). Neurobiological and Systemic Effects of Chronic Stress. Chronic Stress.

[4] He G., Chen T., Huang L., Zhang Y., Feng Y., Qu S., Yin X., Liang L., Yan J., Liu W. (2022). Tremella fuciformis polysaccharide reduces obesity in high-fat diet-fed mice by modulation of gut microbiota. Front. Microbiol..

[5] Park H.J., Shim H.S., Ahn Y.H., Kim K.S., Park K.J., Choi W.K., Ha H.C., Kang J.I., Kim T.S., Yeo I.H. (2012). Tremella fuciformis enhances the neurite outgrowth of PC12 cells and restores trimethyltin-induced impairment of memory in rats via activation of CREB transcription and cholinergic systems. Behav. Brain Res..

[6] Moon G., Rustamov N., Park J., Park H., Park K., Choi E.H., Roh Y.S. (2025). Anti-Stress Effects of Tremella fuciformis Berk. Enzymatic Extracts: A Preclinical Study. Nutrients.

[7] Vahratian A., Blumberg S.J., Terlizzi E.P., Schiller J.S. (2021). Symptoms of Anxiety or Depressive Disorder and Use of Mental Health Care Among Adults During the COVID-19 Pandemic—United States, August 2020–February 2021. MMWR. Morb. Mortal. Wkly. Rep..

[8] Khawam E.A., Laurencic G., Malone D.A. (2006). Side effects of antidepressants: An overview. Clevel. Clin. J. Med..

[9] Pillinger T., Howes O.D., Correll C.U., Leucht S., Huhn M., Schneider-Thoma J., Gaughran F., Jauhar S., McGuire P.K., Taylor D.M. (2023). Antidepressant and antipsychotic side-effects and personalised prescribing: A systematic review and digital tool development. Lancet Psychiatry.

[10] Chauhan S., Tiwari A., Verma A., Padhan P.K., Verma S., Gupta P.C. (2024). Exploring the Potential of Saffron as a Therapeutic Agent in Depression Treatment: A Comparative Review. Yale J. Biol. Med..

[11] Zhao T., Tang H., Xie L., Zheng Y., Ma Z., Sun Q., Li X. (2019). Scutellaria baicalensis Georgi. (Lamiaceae): A review of its traditional uses, botany, phytochemistry, pharmacology and toxicology. J. Pharm. Pharmacol..

[12] Li L., Gao H., Lou K., Luo H., Hao S., Yuan J., Liu Z., Dong R. (2021). Safety, tolerability, and pharmacokinetics of oral baicalein tablets in healthy Chinese subjects: A single-center, randomized, double-blind, placebo-controlled multiple-ascending-dose study. Clin. Transl. Sci..

[13] Dormal V., Suchareau M., Copine S., Simar L., Deldicque L. (2025). The Effects of Combined Scutellaria and Saffron Supplementation on Mood Regulation in Participants with Mild-to-Moderate Depressive Symptoms: A Randomized, Double-Blind, Placebo-Controlled Study. Nutrients.

[14] Yuan K., Zhu S., Wang H., Chen J., Zhang X., Xu P., Xie Y., Zhu X., Zhu W., Sun W. (2021). Association between malnutrition and long-term mortality in older adults with ischemic stroke. Clin. Nutr..

[15] Cederholm T., Barazzoni R., Austin P., Ballmer P., Biolo G., Bischoff S.C., Compher C., Correia I., Higashiguchi T., Holst M. (2017). ESPEN guidelines on definitions and terminology of clinical nutrition. Clin. Nutr..

[16] McKenna S.P., Heaney A. (2020). Composite outcome measurement in clinical research: The triumph of illusion over reality?. J. Med. Econ..

[17] Marra M., Sammarco R., De Lorenzo A., Iellamo F., Siervo M., Pietrobelli A., Donini L.M., Santarpia L., Cataldi M., Pasanisi F. (2019). Assessment of body composition in health and disease using bioelectrical impedance analysis (bia) and dual energy x-ray absorptiometry (dxa): A critical overview. Contrast Media Mol. Imaging.

[18] Dal Bello S., Ceccarelli L., Tereshko Y., Gigli G.L., D’Anna L., Valente M., Merlino G. (2025). Prognostic Impact of Malnutrition Evaluated via Bioelectrical Impedance Vector Analysis (BIVA) in Acute Ischemic Stroke: Findings from an Inverse Probability Weighting Analysis. Nutrients.

[19] Lu W., Zhang T., Li M., Zhang J., Liu N., Yang L., Huang G. (2025). Clinical efficacy and potential mechanisms of acupuncture for Parkinson’s disease: The role of GABAergic signaling. Front. Neurosci..

[20] Zhang T.M., Yu S.Y., Guo P., Du Y., Hu Y., Piao Y.S., Zuo L.J., Lian T.H., Wang R.D., Yu Q.J. (2016). Nonmotor symptoms in patients with Parkinson disease: A crosssectional obser-vational study. Medicine.

[21] Tarakad A., Jankovic J. (2017). Anosmia and Ageusia in Parkinson’s Disease. Int. Rev. Neurobiol..

[22] Cecchini M.P., Fasano A., Boschi F., Osculati F., Tinazzi M. (2015). Taste in Parkinson’s disease. J. Neurol..

[23] Duffy V.B., Backstrand J.R., Ferris A.M. (1995). Olfactory Dysfunction and Related Nutritional Risk in Free-Living, Elderly Women. J. Am. Diet. Assoc..

[24] Barichella M., Cereda E., Cassani E., Pinelli G., Iorio L., Ferri V., Privitera G., Pasqua M., Valentino A., Monajemi F. (2017). Dietary habits and neurological features of Parkinson’s disease patients: Implications for practice. Clin. Nutr..

[25] Alia S., Andrenelli E., Di Paolo A., Membrino V., Mazzanti L., Capecci M., Vignini A., Fabri M., Ceravolo M.G. (2025). Chemosensory Impairments and Their Impact on Nutrition in Parkinson’s Disease: A Narrative Literature Review. Nutrients.

[26] Ashina M., Buse D.C., Ashina H., Pozo-Rosich P., Peres M.F.P., Lee M.J., Terwindt G.M., Halker Singh R., Tassorelli C., Do T.P. (2021). Migraine: Integrated approaches to clinical management and emerging treatments. Lancet.

[27] Tu Y.H., Chang C.M., Yang C.C., Tsai I.J., Chou Y.C., Yang C.P. (2025). Dietary Patterns and Migraine: Insights and Impact. Nutrients.

[28] Crowe F.L., Steur M., Allen N.E., Appleby P.N., Travis R.C., Key T.J. (2011). Plasma concentrations of 25-hydroxyvitamin D in meat eaters, fish eaters, vegetarians and vegans: Results from the EPIC-Oxford study. Public Health Nutr..

[29] Hindiyeh N.A., Zhang N., Farrar M., Banerhee P., Lombard L., Aurora S.K. (2020). The Role of Diet and Nutrition in Migraine Triggers and Treatment: A Systematic Literature Review. Headache.

[30] Trichopoulou A., Costacou T., Bamia C., Trichopoulos D. (2003). Adherence to a Mediterranean diet and survival in a Greek population. N. Engl. J. Med..

[31] Perrone L., Grant W.B. (2015). Observational and ecological studies of dietary advanced glycation end products in national diets and Alzheimer’s disease incidence and prevalence. J. Alzheimer’s Dis..

[32] Kim S.G., Keum M., Choe Y.M., Suh G.H., Lee B.C., Kim H.S., Lee J.H., Hwang J., Yi D., Kim J.W. (2025). Selenium and Episodic Memory: The Moderating Role of Apolipoprotein E epsilon4. Nutrients.

[33] Gustavsson A., Norton N., Fast T., Frolich L., Georges J., Holzapfel D., Kirabali T., Krolak-Salmon P., Rossini P.M., Ferretti M.T. (2023). Global estimates on the number of persons across the Alzheimer’s disease continuum. Alzheimer’s Dement..

[34] Roda A.R., Serra-Mir G., Montoliu-Gaya L., Tiessler L., Villegas S. (2022). Amyloid-beta peptide and tau protein crosstalk in Alzheimer’s disease. Neural Regen. Res..

[35] Misrani A., Tabassum S., Yang L. (2021). Mitochondrial Dysfunction and Oxidative Stress in Alzheimer’s Disease. Front. Aging Neurosci..

[36] Sbai O., Djelloul M., Auletta A., Ieraci A., Vascotto C., Perrone L. (2022). RAGE-TXNIP axis drives inflammation in Alzheimer’s by targeting Aβ to mitochondria in microglia. Cell Death Dis..

[37] Sbai O., Bazzani V., Tapaswi S., McHale J., Vascotto C., Perrone L. (2023). Is Drp1 a link between mitochondrial dysfunction and inflammation in Alzheimer’s disease?. Front. Mol. Neurosci..

[38] Rezaee N., Hone E., Sohrabi H., Abdulraheem R., Johnson S.K., Gunzburg S., Martins R.N., Fernando W. (2025). Investigating the Impact of Sorghum on Tau Protein Phosphorylation and Mitochondrial Dysfunction Modulation in Alzheimer’s Disease: An In Vitro Study. Nutrents.

[39] Dowis K., Banga S. (2021). The Potential Health Benefits of the Ketogenic Diet: A Narrative Review. Nutrients.

[40] Phillips M.C.L., Deprez L.M., Mortimer G.M.N., Murtagh D.K.J., McCoy S., Mylchreest R., Gilbertson L.J., Clark K.M., Simpson P.V., McManus E.J. (2021). Randomized crossover trial of a modified ketogenic diet in Alzheimer’s disease. Alzheimer’s Res. Ther..

[41] Bohnen J.L.B., Albin R.L., Bohnen N.I. (2023). Ketogenic interventions in mild cognitive impairment, Alzheimer’s disease, and Parkinson’s disease: A systematic review and critical appraisal. Front. Neurol..

[42] Gentili V., Schiuma G., Dilliraj L.N., Beltrami S., Rizzo S., Lara D., Giovannini P.P., Marti M., Bortolotti D., Trapella C. (2024). DAG-MAG-BetaHB: A Novel Ketone Diester Modulates NLRP3 Inflammasome Activation in Microglial Cells in Response to Beta-Amyloid and Low Glucose AD-like Conditions. Nutrients.

